# An investigation on some toxic effects of pyriproxyfen in adult male mice

**DOI:** 10.22038/ijbms.2019.33825.8051

**Published:** 2019-09

**Authors:** Amna Shahid, Syeda Durr-e- Shahwar Zaidi, Haroon Akbar, Sania Saeed

**Affiliations:** 1 Department of Zoology, Government College University, Katchery Road, Lahore, Pakistan; 2 Department of Parasitology, University of Veterinary and Animal Sciences, Lahore, Pakistan

**Keywords:** Endocrine disruptor, Histopathology, Leydig cells, Pyriproxyfen, Reproductive toxicity, Seminiferous tubules

## Abstract

**Objective(s):**

Pyriproxyfen as an insect growth regulator is widely used globally for pest management. There are reports on adverse effects of insecticides such as organ toxicity, endocrine disruptions, and teratogenicity in animals and humans. We aimed to investigate reproductive toxicity of pyriproxyfen in adult male mice.

**Materials and Methods:**

48 male Swiss albino mice were divided into eight groups and received the different 1200, 600, 320, 200, 100, 40, 20, 0 mg/kg/day doses orally, and body weights were accessed for 28 consecutive days. In the end, mice were sacrificed, testes were dissected and weighed. Probable testicular tissue alterations were examined by histopathological studies. In addition, the diameter of seminiferous tubules and Leydig cells distribution were assessed in all experimental and control groups.

**Results:**

Pyriproxyfen treatment caused significant (*P*<0.05) reduction in body and organ weights in mice. However, the shrinkage and displacement of seminiferous tubules, reduced lumen diameter, and vacuolization occurred in seminiferous tubules in higher doses exposed animals in comparison to controls. The relative testis weights, mean diameter of seminiferous tubules, and Leydig cells distribution remained unchanged at low doses.

**Conclusion:**

These findings reveal that pyriproxyfen caused reduction in body weight gain as well as damage to the testicular architecture in mice and thus may potentially interfere with spermatogenesis. Findings in an outbred strain of mice can be extrapolated fairly reliably to the human model. The chemical can thus be further exploited to study its effects on impairment of fertility and as an endocrine disruptor.

## Introduction

Insecticides are extensively used in agricultural practices to obtain higher crop yield and control insects, animals, and vector borne diseases. Previous studies have shown that insecticides may cause hazardous effects due to their potential toxicity to both animals and humans (1, 2). Globally, over 800 active ingredients of insecticides are sold in tens of thousands of formulations (3) and approximately 2 million tons of pesticides are utilized annually (4). According to WHO reports, there are more than 3 million human pesticide poisonings with about 250,000 deaths per year worldwide (5). 

Pesticides are classified according to their target site, modes or periods of action, and chemical compositions (6). Chemical compounds that interfere and induce changes in the growth and developmental process of insects are known as insect growth regulators (IGRs). They can be classified according to their mode of action, i.e., chitin synthesis inhibitors (disrupts the cuticle formation) and juvenile hormones (mimics the action of insect hormones). Some of the most important IGRs include diflubenzuron, lufenuron, buprofezin, methoprene, kinoprene, hydroprene, fenoxycarb, and pyriproxyfen (7). 

Pyriproxyfen, 2-{1-methyl-2-(4-phenoxyphenoxy) ethoxy} was first manufactured by Sumitomo Chemical Company, in 1990. It is pyridine based, an aromatic, non-terpenoidal potent inhibitor of embryogenesis and adult emergence in insects (8). As a juvenile hormone analog, pyriproxyfen acts as an endocrine disruptor, which alters the endocrine system and ultimately causes injurious health problems in target organisms (9). 

Pyriproxyfen is widely used to combat arthropods, including insects, weeds, and annual grasses. It is recommended by WHO to primarily be used in drinking water for vector control and public health programs (10, 11). It has high stability in the environment, and persistence via food chain causes detrimental effects on non-target species (12). In the previous report, the neurodevelopmental toxicity at different doses of pyriproxyfen (0, 100, 300, and 1000 mg/kg/day) in rat pups was evaluated, and the results revealed arhinencephaly and reduction in brain weight in treated pups as compared to control pups (13). The continuous use of insecticides in some countries can lead to interference with endocrine homeostasis, resulting in the impairment of the male reproductive system (14, 15). Therefore, the aim of the present study was to investigate the toxic effects of pyriproxyfen on overall body weight gain of the Swiss albino mice and histopathological changes, particularly in the testis of male mice.

## Materials and Methods

***Animals***

Forty-eight adult male Swiss albino mice (*Mus musculus*) weighing between 19 and 32 g (7–8 weeks old) were obtained from the Animal House in the Department of Zoology, Government College University, Lahore, Pakistan. The animals were maintained in the experimental room for acclimatization (1 week) at the proper temperature (25±2 ^°^C) and 12 hr light/dark cycles. The mice were housed in special polycarbonate cages and fed laboratory diet and water *ad libitum*. All experimental procedures were carried out with the permission and in accordance with the rules of Ethical Committee for the Treatment of Animals, Government College University of Lahore.

***Chemicals***

Predator 0.5 (pyriproxyfen) Water-Dispersible Granules (WDG) was obtained from Evyol Chemicals group, Lahore, Pakistan. Different grades of alcohol, formaldehyde 37%, xylene, Hematoxylin, and Eosine stains were supplied by Sigma-Aldrich (Germany). Ketamine and xylazine were purchased from Elite Pharma (Pvt) Ltd. Lahore, Pakistan. 

***Dosage and treatment***

The mice were divided into 8 groups, each comprised six mice, namely I, II, III, IV, V, VI, VII, and control (untreated group). The control group did not receive pyriproxyfen, while the experimental group received dosage of pyriproxyfen 1200, 600, 320, 200, 100, 40, and 20 mg/kg BW per day. The dose was administered orally via gavage for 28 consecutive days. The animals were weighed daily during the experimental period.

***Absolute and relative organ weights***

The mice were euthanized by intraperitoneal (IP) injections with ketamine (100 mg/kg) and xylazine (10 mg/kg); organs (testis) were removed, cleaned, and weighed. The absolute and relative weights of the right and left testis were calculated.

$\mathrm{Relative organ weight}=\frac{\mathrm{Absolute organ weight}(g)}{\mathrm{Body weight of mice}(g)}\times 100$      (16)

***Histopathological analyses***

The tissues were fixed in 10% formalin solution for 24 hr and processed through the paraffin embedding technique and cut at 3–5 μm thickness (17). The tissue sections were stained using Hematoxylin and Eosin (H&E) (18). The slides were observed under the microscope (Olympus CX31 Binocular), and histopathological changes were observed in the tissues with the help of an Olympus digital camera.

***Morphometric parameters***

For assessment of morphometric parameters, the tissue sections were studied using 100X and 400X magnifications. The diameter of seminiferous tubules was obtained by random selection of 100 cross sections of the seminiferous tubules from each mice. The seminiferous tubule diameters were measured across major and minor axis using an ocular micrometer with light microscopy, and their means were obtained (19). The Leydig cell counts (in one µm^2^ of interstitial tissue) were also done in tissue sections of all treated and control groups.

**Figure 1:**
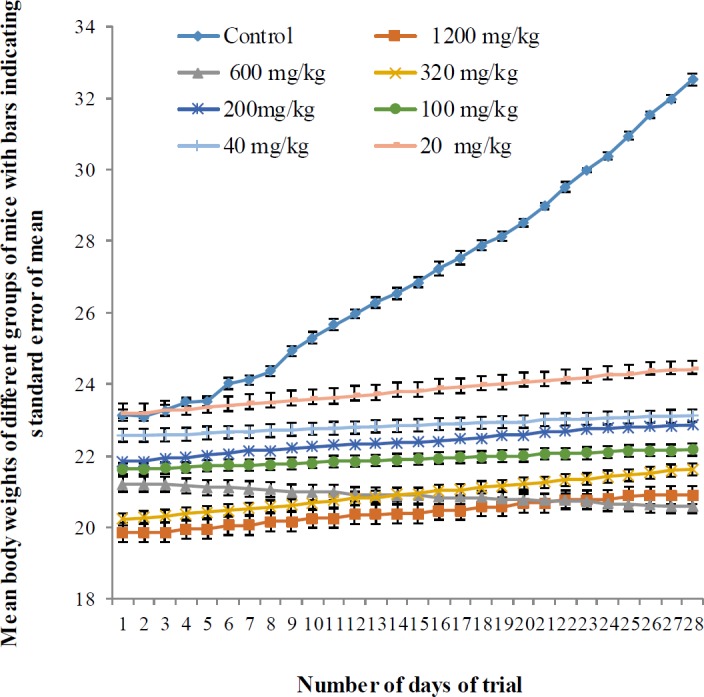
Mean body weights of mice in control and pyriproxyfen treated groups during 28 consecutive days

**Figure 2:**
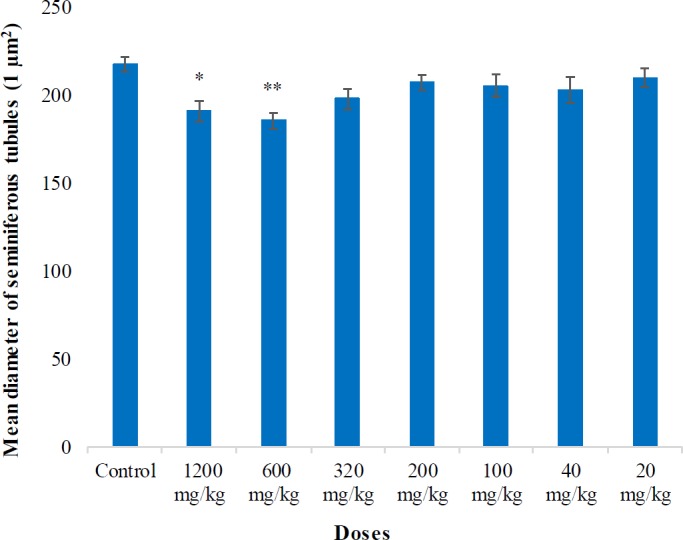
Mean diameter of seminiferous tubules (1 µm^2^) in the testis of mice in control and pyriproxyfen treated groups. *: a significant difference between control and 1200 mg/kg groups (*P*<0.05). **: a significant difference between control and treated (600 mg/kg) groups (*P*<0.01)

**Figure 3:**
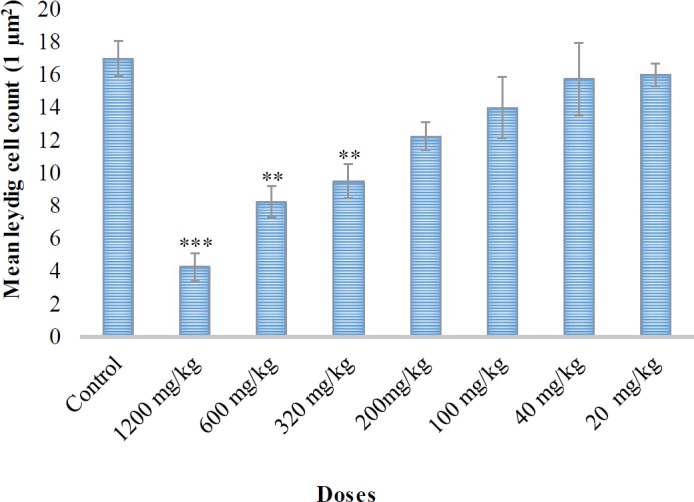
Mean Leydig cell count (1 µm^2^) in the testis of mice in control and pyriproxyfen treated groups. ***: a significant difference between control and 1200 mg/kg groups (*P*<0.001). **: a significant difference between control and treated (600 mg/kg and 320 mg/kg) groups (*P*<0.01)

**Figure 4:**
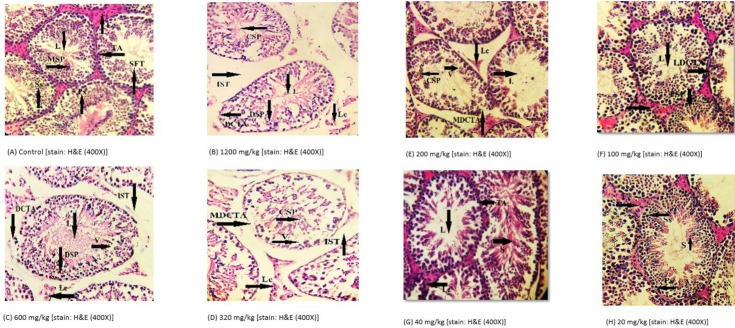
(A) Control group mice testes after 28 days of experimental period showing normal testicular tissue with mature spermatozoa (MSP), normal seminiferous tubules (SFT) with normal basement membrane tunica albuginea (TA), reduced lumen (L), increased number of Leydig cells (Lc) and primary spermatozoa (Ps) and secondary spermatozoa (Ss) were also present. (B) Pyriproxyfen treated group (1200 mg/kg) showing reduced lumen (L), more intra seminiferous tubular space (IST), more clumping of spermatozoa (CSP), degenerative changes in tunica albuginea (DGTA), more degenerative spermatozoa (DSP), and Leydig cells (Lc) were absent. Figure (C) Pyriproxyfen treated group (600 mg/kg) showing reduced lumen (L), less intra seminiferous tubular space (IST), degenerative changes in tunica albuginea (DTA), clumped spermatozoa (CSP), vacuolization (V), and degenerative Leydig cells (Lc) were present. Figure (D) Pyriproxyfen treated group (320 mg/kg) showing less, seminiferous tubular space (IST), moderate degenerative changes in tunica albuginea (MDCTA), lower number of Leydig cells (Lc), more vacuolization (V). Figure (E) Pyriproxyfen treated group (200 mg/kg) showing mild degenerative changes in tunica albuginea (MDCTA), presence of Leydig cells (Lc), less clumping of spermatozoa (CSP) and increase in the lumen (L). Figure (F) Pyriproxyfen treated group (100 mg/kg) showing lumen (L), primary spermatozoa (Ps), less degenerative changes in the tunica albuginea (LDCTA), and Leydig cells (Lc) were present. Figure (G) Pyriproxyfen treated group (40 mg/kg) showing higher number of sperms (S), and Leydig cells (Lc) with normal tunica albuginea (TA). Figure (H) Pyriproxyfen treated group (20 mg/kg) showing primary spermatozoa (PS), secondary spermatozoa (Ss), increased number of sperms (S), and increased number of Leydig cells (Lc)

**Table 1:** Body, testis, and relative testis weights of different groups

	Initial body weights **(g)**	Final body weights **(g)**	Right testis weights **(g)**	Relative right testis weights **(g)**	Left testis weights **(g)**	Relative left testis weights **(g)**
Control	23.13 ± 0.49	32.51 ± 1.46	0.085 ± 0.44	0.261 ± 0.02	0.084 ± 0.41	0.258 ± 0.02
1200 mg/kg	19.85 ± 0.91***	20.90 ± 0.57***	0.064 ± 0.40*	0.306 ± 0.01	0.064 ± 0.57*	0.306 ± 0.01
600 mg/kg	21.2 ± 0.70***	20.58 ± 1.04***	0.069 ± 0.56*	0.335 ± 0.01	0.069 ± 0.45*	0.335 ± 0.01
320 mg/kg	20.21 ± 1.14***	21.61 ± 1.02***	0.074 ± 0.41	0.342 ± 0.02	0.074 ± 0.27	0.342 ± 0.001
200 mg/kg	21.85 ± 0.95***	22.85 ± 1.30***	0.076 ± 0.38	0.332 ± 0.002	0.074 ± 0.37	0.323 ± 0.009
100 mg/kg	21.63 ± 0.30***	22.16 ± 0.76***	0.078 ± 0.32*	0.351 ± 0.007	0.082 ± 0.41	0.370 ± 0.002
40 mg/kg	22.56 ± 0.26	23.11 ± 0.45***	0.083 ± 0.26	0.359 ± 0.001	0.087 ± 0.37	0.376 ± 0.001
20 mg/kg	23.18 ± 0.41*	24.40 ± 0.40***	0.083 ± 0.28	0.340 ± 0.009^#^	0.085 ± 0.55	0.348 ± 0.006^#^

Values are expressed as mean ± SEM **(n=6)**. One-way ANOVA followed by Tukey’s ***post hoc*** test, *** *P*<0.001, * *P*<0.05 and # (not significant) represents comparison to control and treated groups

**Table 2:** Determination of structural changes by histopathological examination of the albino mice testis in the different treatment groups

Groups	Doses mg/kg	Spermatids	Spermatozoa	Leydig cells	Sperm density	Degenerative changes in tunica albuginea	Intratubular space
Control		**++++**	**++++**	**++++**	**++++**	**-**	**-**
I	1200	**+**	**+**	**-**	**+**	**++++**	**++++**
II	600	**+**	**+**	**+**	**+**	**++++**	**++++**
III	320	**++**	**++**	**+**	**+**	**+++**	**+++**
IV	200	**++**	**++**	**++**	**+**	**++**	**++**
V	100	**+++**	**+++**	**+++**	** ++**	**++**	**++**
VI	40	**++++**	**++++**	**++++**	** ++**	**+**	**+**
VII	20	**++++**	**++++**	**++++**	** ++++**	**+**	**+**

++++ indicates extremely severe, +++ indicates severe, ++ indicates moderate, + indicates mild, - indicates absent

**Table 3:** Mean diameter of seminiferous tubules (1 µm^2^) in testes in different groups

Groups	Doses (mg/kg)	Mean diameter of seminiferous tubules (1 µm^2^) X ± SEM
Control	0	217.86 ± 4.14
I	1200	191.10 ±5.94*
II	600	185.68 ± 4.52**
III	320	198.11 ± 5.94
IV	200	207.43 ± 4.43
V	100	205.80± 6.29
VI	40	203.33 ± 7.43
VII	20	210.33 ± 5.24

* *P*<0.05 and **P<0.01 indicate significant difference between treated mice groups (1200 and 600 mg/kg) and control group.

**Table 4:** Mean distribution of Leydig cells (1 µm^2^) in different groups

Groups	Doses (mg/kg)	Mean **Leydig** cells count (1 µm^2^) (X ± SEM)
Control	0	17.01 ± 1.08
I	1200	4.25 ± 0.85***
II	600	8.25 ± 0.94**
III	320	9.50 ± 1.04**
IV	200	12.50 ± 0.85
V	100	14.31 ± 1.87
VI	40	15.75 ± 2.21
VII	20	16.23 ± 0.70

** *P*<0.01 indicates significant difference between control and treated groups (600 and 320 mg/kg). *** *P*<0.001 shows significant difference between the treated group (1200 mg/kg) in comparison with controls.

***Statistical analysis***

All data were presented as Mean±SEM; analysis of variance (one way ANOVA) was used followed by Tukey’s multiple comparisons test as *post hoc* for inter group organ weights, relative organ weights, mean tubular diameter and Leydig cell count comparison. In this study, the *P*-value of less than 0.05 was considered significant using SPSS version 17. Graph Pad Prism, version 0.5 (two way ANOVA followed by Bonferroni *post hoc* analysis) was used for comparison of body weights in all treated and control groups.

## Results

***Body and organ weights***

The behavioral changes, clinical signs of systemic toxicity, and mortality were monitored during the experimental period. There were no morbidity, mortality, or behavioral changes recorded during the specific time period. In addition, there were no obvious differences in water and food consumption between the experimental groups. 

The mean initial body weight of the control group was 23.13±0.49 g, and the final body weight was 32.51± 1.46 g after the 28 day experimental period. The mean body weights of mice in all experimental groups were recorded on a regular basis whereas in group I (1200 mg/kg), the initial and final body weights were 21.85±0.95 g and 22.85±1.30 g. Overall, the results indicated that Pyriproxyfen treatment caused a significant decrease (*P*<0.001) in body weights of mice in all treated groups as compared to controls (Table 1 and Figure 1). 

In addition, pyriproxyfen treatment resulted in more testis weight reduction compared with the untreated (control) group. The weights of right and left testes of mice in the control group were measured as 0.085±0.44 g and 0.084±0.41 g, respectively while the weights of right and left testis were 0.064±0.40 g and 0.064±0.57 g, respectively in the experimental group (1200 mg/kg). There was also a significant decrease (*P*<0.05) in the absolute testis weights of treated mice in comparison with control mice (Table 1). 

***Relative organ weights***

The differences in the relative weights of right and left testis in treated groups were statistically insignificant compared with the control group (*P*>0.05) as shown in Table 1. 

***Histopathological abnormalities in testicular structure***

Histopathological examinations of testicular architecture showed severe degenerative changes in tunica albuginea at high doses (1200, 600, 320, 200 mg/kg) as compared with the control group. Sperm count was decreased at high doses, and vacuolization was also clearly visible in the lumen of seminiferous tubules. There were increases in intratubular space and clumped spermatozoa, and widening of lumen diameter at high doses of pyriproxyfen. Degeneration and scattering of spermatids were also observed in pyriproxyfen treated mice testes more than in the control group (Table 2 and Figures 4 A-H). 

***Morphometric analysis***

There were no significant differences (*P*>0.05) in the mean diameters of seminiferous tubules (μm^2^) between treated groups (320, 200, 100, 40, and 20 mg/kg) and the control group (Figure 2). The reduction in Leydig cell count (µm^2^) was also observed on day 28 after oral administration of pyriproxyfen with a significant difference between control vs 1200 mg/kg (*P*<0.001) and control vs 600 and 320 mg/kg groups (*P*<0.01), (Figure 3). 

## Discussion

The continuous and/or inappropriate exposure to synthetic pesticides could be a possible risk factor for increased male infertility and reduced sperm counts in humans. The hazardous effects of pesticides in the environment first received attention in the 1960s worldwide. Furthermore, recent studies suggest that long-term and low exposure to these chemical substances are mainly associated with various human health problems such as cancer, endocrine disruption, immuno-suppression, and reproductive dysfunction (20). 

In toxicological studies, the body, organs, and relative organ weights are specifically used as markers for organ toxicity (21, 22). The present study revealed a significant decrease (*P*<0.001) in body weights of mice in all treated groups as compared to control mice (Table 1 and Figure 1). In addition, there was a significant reduction (*P*<0.05) in absolute and relative weights of the right and left testes of treated mice (Table 1). In line with our results, Mehrnoush *et al*. (2013) documented a significant decrease in body weights of rats in pyriproxyfen treated groups (1000, 2000, and 4000 mg/kg BW) (12). Another previous study that evaluated the effects of pyriproxyfen on dogs and rats showed a reduction in the seminal vesicle, prostate, and androgen sensitive organ weights (23). Furthermore, research showed (24), significant decrease (*P*<0.05) of body weight in rats exposed to dimethoate as compared with control rats. It has been reported that insecticidal products such as cypermethrin alone or in combination with sodium fluoride, malathion, solignum (a commercial permethrin-containing wood preservative known as SOL), and chlorpyrifos caused , reduction in body and testicular weights of rodents (16, 25-27). 

In comparison with our results, a study (28) speculated slight decrease in body weights of male rats treated with diflubenzuron but significant decrease in the absolute and relative weights of the rats’ testes at a lower dose (2 mg/kg) of diflubenzuron. In another study, the male rats exposed to β-cyfluthrin (15.2 and 7.6 mg/kg) exhibited a reduction in body weights significantly, but there was no significant difference in testis weights of mice in any experimental groups (29). In addition, researchers (30) also reported an increase in both body and testicular weights in rats orally fed permethrin-containing diet. The testis weight is mainly dependent on the number of differentiated spermatocytes and reduction in the size of seminiferous tubules. Elongated spermatids and decreased population of germ cells tend to decrease the weight of testis (31). 

Histopathology is considered the most appropriate parameter for the evaluation of toxicity in the male reproductive system (32, 33). The impaired fertility may be due to the direct action of insecticide on the testis and disruption of the androgenic activity. A toxic substance that acts directly on anterior pituitary gland could influence the testis indirectly and ultimately alter sexual activity (34). Our results indicated testicular abnormalities including displacement and shrinkage of seminiferous tubules, vacuolization, reduced lumen diameter and sperm count, absence of Leydig cells, degenerative changes in tunica albuginea, and increased interstitial space in mice that received higher doses of pyriproxyfen (1200, 600, and 320 mg/kg) (Figures 1 A-B-C). Overall, the mice treated with the lowest doses, i.e., 200, 100, 40, and 20 mg/kg pyriproxyfen showed less degenerative changes in testicular architecture (Figures 1 D-E-F-G). 

In addition, a study revealed decreased spermatogenic cells, degeneration in Leydig cells, and abnormal seminiferous tubules having vacuolization in rats inhaling pyrethroids (35). In accordance to our present results (Table 2), many other authors investigated the testicular histopathology that revealed inhibition of spermatogenesis and mild to severe degenerative changes in seminiferous tubules in albino rats exposed to chlorpyrifos (36, 37) and in lambda-cyhalothrin treated male mice (38). Furthermore, researchers documented (39) the damage in the germinal epithelium, presence of apoptotic cells, blockage of spermatogenesis process, and wide lumen without the spermatozoids in the testes of lambda-cyhalothrin treated rats. 

In addition, similar findings by another study (40) showed that diazinon treatment might degenerate seminiferous tubules, thereby inhibit spermatogenesis. In the present study, there was a significant reduction (*P*<0.001) in the mean number of Leydig cells in the treated group (1200 mg/kg) as compared to controls (Figure 3). Similar to our results, the number of Leydig cells in mice testis were significantly reduced in a dose-dependent manner (40). A histopathological study of the testis of male Sprague-Dawley rats treated with fenitrothion revealed observations such as degeneration of germ cells and Leydig cells, increase in interstitial space, disorganization of spermatogonia in the seminiferous tubules, and necrotic changes in the seminiferous tubules (41). 

In our findings, the diameters of seminiferous tubules in treated mice at 1200 mg/kg were significantly reduced (*P*<0.05) as compared to control mice (Figure 2). These results are in agreement with the findings by Najafi *et al*. (2010)**, **who described that imidacloprid treatment in adult male rats exhibited a significant reduction in the number of Leydig cells, Sertoli cell dysfunction, and diameter of seminiferous tubules (42). Similar histopathological disturbances were observed in the rodent testicular structure, i.e., reduced diameter of seminiferous tubules, disruption of germ cells, reduction in Leydig cell size, and number and absence of sperms in the lumen, when exposed to dimethoate and other organophosphates (43–47). In contrast to our findings, there were no variations in surface area and diameter of seminiferous tubules in the testis of rats treated with cadmium and diazinon (48). 

## Conclusion

The results obtained from the present study indicated that different doses of pyriproxyfen lead to disruption of spermatogenesis in seminiferous tubules. Hence, our study suggested that pyriproxyfen usage must be cautiously done with the consideration of different hazardous levels. However, to achieve this information, the molecular mechanism of the interaction of pyriproxyfen with germ cells should be explored in future studies. 
